# Multidimensional Emotional Disorder Inventory: reliability and validity in a Colombian non-clinical sample

**DOI:** 10.1186/s41155-024-00304-3

**Published:** 2024-07-10

**Authors:** Santiago Zarate-Guerrero, Leonidas Castro-Camacho, Yvonne Gomez-Maquet, Johanna Duran-Molina

**Affiliations:** 1 Departamento de Psicología, Facultad de Ciencias Sociales, Universidad de los Andes, Bogotá, Colombia; 2 Facultad de Ciencias Sociales y Humanas, Fundación Universitaria del Area Andina, Bogotá, Colombia

**Keywords:** Dimensional assessment, Emotional Disorders, Transdiagnostic measures, Cultural adaptation

## Abstract

**Background:**

Contemporary diagnostic frameworks in the realm of mental health have garnered criticism due to their categorical paradigm. Given the propensity of emotional disorders to manifest overlapping features, these frameworks fall short in comprehensively encapsulating their intricate nature. As a strategic response, Brown and Barlow introduced an innovative composite approach, amalgamating dimensions and categorical classifications, to adress this concern. Their strategic implementation hinged on the Multidimensional Emotional Disorder Inventory (MEDI), a transdiagnostic self-report instrument. Objective: this study undertakes the task of refining and validating the applicability of the MEDI within a non-clinical sample of Colombian university students (*n* = 808).

**Methods:**

This study employed Exploratory Structural Equation Modeling (ESEM) to explore the structure of the measure. Results: ESEM suggested that the 8-dimension model with 48 items was the best-fitting solution, aligning with most dimensions identified by the original MEDI validation. Reliability was adequate for almost all dimensions (α: 0.69 – 0.92). An 8-dimension model with 48 items emerged as the most fitting solution, aligning with most dimensions identified by the original MEDI validation.

**Conclusion:**

The ensuing validation and contextual adaptation of the MEDI for use in the Colombian population augments the transdiagnostic evaluation of emotional disorders, with potential implications for enhanced stratification of targeted therapeutic interventions. By optimizing the assessment of both dimensional and cross-diagnostic paradigms, the MEDI portends a noteworthy impact in realms encompassing both academic inquiry and clinical practice.

## Introduction

The current diagnostic systems for classifying psychopathology are categorical, and both main international systems in use, the Diagnostic and Statistical Manual of Mental Disorders (DSM-5 TR) (American Psychiatric Association, 2022), and the International Classification of Disease (ICD-11) (World Health Organization, 2018), have several limitations. One of these limitations is the overlap between categories, leading to high comorbidity between categories, especially in the context of emotional disorders (including Anxiety Disorders, Unipolar Depression, Post Traumatic Stress Disorder, Obsessive and Compulsive Disorder) (Broman-Fulks et al., 2010). Furthermore, the categorical classification systems primarily focus on symptoms rather than underlying core or maintaining processes (Brown & Barlow, 2005). Despite both DSM-5 and ICD-11 helping mental health professionals differentiate one disorder from another, and many of them being required to use diagnostic codes for health insurance policies, it is evident that categorical systems fall short in providing information about treatments (Rosellini et al., 2015). As a result, there has been a shift in the assessment of emotional disorders, moving away from discrete categorical constructs towards a dimensional approach (Emmert-Aronson, 2016). Some of the initiatives in psychopathology aimed at addressing this change include the Research Domain Criteria (RDoC), which is a transdiagnostic approach incorporating dimensions of psychological processes across different domains (Insel et al., 2010) and the Hierarchical Taxonomy of Psychopathology (HiTOP), a quantitative and dimensional classification system based on a multilevel organization (Kotov et al., 2017). Both have demonstrated strong theoretical value but limited clinical utility. First because RDoC defines its domains mainly by biological processes (Rosellini & Brown, 2019). Second, despite HiTOP dimensions being more descriptive and specific than those in the RDoC, benefiting in a clinical scenario would require several specific questionnaires to evaluate each dimension, leading to patients burnout and less utility in clinical decision-making for treatment assignment (Zarate‐Guerrero et al., 2022).

Considering the necessity to create a dimensional assessment framework in the field of emotional disorders, defined as any psychological disorder that meets these three criteria (1) experience of frequent and intense experience of negative emotions, (2) aversive reaction to the emotional experiences accompanied by a diminished sense of control and negative appraisal of emotions, (3) engagement in emotional avoidance reactions (Bullis et al., 2019), Brown and Barlow (2009) developed a hybrid dimensional – categorical approach that includes a series of factors common to emotional disorders. They argued that based on the scores of such factors, a disitnctive profile for each patient would emerge. This unique profile would be closer or distant to the diagnostic categories provided by DSM or ICD categories. Brown and Barlow (2009) defined ten unique factors: (1) neuroticism, anxiety or behavioral inhibition, neurotic temperament (NT), (2) behavioral activation or positive affect, positive temperament (PT), (3) depressed mood (DM), (4) mania (MA), (5) autonomic arousal (AA), (6) somatic anxiety (SOM), (7) social anxiety (SEC), (8) intrusive cognitions (IC), (9) traumatic re-experiencing and dissociation (TRM), and (10) avoidance (AVD). The main purpose of this approach was to increase the reliability and validity of the established diagnosis by facilitating differential diagnosis and reducing comorbidity rates by focusing on dimensional aspects of individuals that share a common diagnosis (Osma et al., 2021). Nonetheless, without a unified instrument that assesses all factors, this approach requires several self-report questionnaires to assess each dimension included in the profile, leading to time and cost-effectiveness issues in clinical practice (Rosellini & Brown, 2019).

Given that the majority of assessment instruments have traditionally prioritized diagnostic categories over transdiagnostic mechanisms, the primary challenge was to develop clinical measures capable of identifying individual transdiagnostic dimensions. There have been some measures developed to address specific transdiagnostic mechanisms, such as Intolerance to Uncertainty (Einstein, 2014; Freeston et al., 1994), Anxiety Sensitivity Index (McNally, 1996; Taylor, 1999), and Ruminative Response Scale (Carleton et al., 2007; Deacon et al., 2003; Nolen-Hoeksema, 1991). However, there are not yet specific instruments addressing most of the transdiagnostic mechanisms in emotional disorders. Therefore, the primary need was still the development and validation of a dimensional instrument capable of capturing most core transdiagnostic dimensions.

The Multidimensional Emotional Diagnostic Inventory (MEDI) was developed not only to assess dimensionally nine of the ten unique factors created by Brown and Barlow (2009), but also to reduce the burden of completing different self-report questionnaires that assess independently assess symptoms and processes related to one or more emotional disorders (Rosellini, 2013; Rosellini & Brown, 2019). The exclusion of the Mania dimension from the MEDI was based on the low rates and severity of manic symptoms, making it impossible to test convergent validity (Rosellini & Brown, 2019). The MEDI assesses nine transdiagnostic dimensions, and the scores are interpreted using profile-dimensional approaches as explained by Rosellini and Brown (2015):

1. Neurotic Temperament: This dimension delves into emotional reactions to minor issues, such as Item 1: "I get upset by trivial things."
2. Positive Temperament: It gauges one's propensity to find humor and positivity in everyday situations, like Item 2: "It doesn't take much to make me laugh."
3. Depressive Mood: This dimension explores feelings of sadness and melancholy, as illustrated by Item 11: "I feel sad and blue."
4. Autonomic Arousal: It evaluates physical symptoms like breathlessness, reflected in Item 4: "I have been experiencing breathlessness."
5. Somatic Anxiety: Concerns related to health and physical well-being, such as Item 19: "I worry about my health," are assessed in this dimension.
6. Social Anxiety: This dimension focuses on discomfort in social situations, as indicated by Item 7: "I am uncomfortable mingling at social events."
7. Intrusive Thoughts: It measures thoughts that might be considered unconventional by others, including Item 5: "Other people would consider some of my thoughts to be odd."
8. Traumatic Re-experiencing: This dimension examines persistent thoughts about distressing experiences, like Item 8: "I cannot stop thinking about horrific things that I have experienced or seen."
9. Avoidance: It assesses how individuals cope with distressing thoughts, feelings, or images, such as Item 9: "I cope with unpleasant thoughts, feelings, or images by trying to distract myself."

Briefly, the hybrid dimensional-categorical approach proposed by Brown and Barlow (2009) differentiates between higher and lower-order dimensions. The former comprises temperament dimensions based on the etiology of emotional disorders (neurotic and positive temperament), while the latter include mood states (depressive mood) and dimensions focusing on anxiety (e.g., somatic anxiety, social evaluation concerns, etc.) and emotion-driven behaviors (avoidance). Thus, the MEDI dimensions also encompass both transdiagnostic mechanisms and transdiagnostic dimensions associated with emotional disorders.

The MEDI holds potential utility for identifying specific phenotypes and vulnerabilities maintaining emotional disorders, thus enabling targeted treatments. This self-reported questionnaire efficiently assesses nine transdiagnostic dimensions, allowing clinicians to obtain an overview of patient functioning across these dimensions. The MEDI provides benefits both in research and clinical settings. From a research standpoint, it promotes the study of a dimensional approach to classifying emotional disorders, facilitating exploration of symptom severity and interference based on shared dimensions rather than specific symptoms or diagnostic criteria. Clinically, it assists clinicians in obtaining information that can be further explored with clinical tools such as functional analysis. The MEDI aids in identifying treatment targets and prioritizing therapeutic objectives. Moreover, it facilitates the measurement of pre- and post-intervention changes in each dimension when treating cases of emotional disorders.

Furthermore, when considering the use of MEDI in a clinical practitioner's context, assessing individual scores can aid in constructing patient profiles that highlight the key mechanisms underpinning emotional disorders. For instance, regardless of whether a patient has received diagnoses such as panic disorder, specific phobias, or social anxiety, if they exhibit elevated levels of avoidance concerning their physical sensations, treatment should be directed toward techniques that specifically address this interoceptive avoidance. In essence, rather than relying solely on categorical diagnoses, identifying the transdiagnostic processes sustaining the disorder (e.g., interoceptive avoidance) enables the development of a more precise and tailored treatment plan (Barlow et al., 2004; Craske, 2017; Gallagher, 2017). This approach not only enhances our comprehension of the factors perpetuating emotional disorders but also addresses comorbidity issues by providing greater insight into the processes at play in each patient. A dimensional classification system has implications for the implementation of transdiagnostic treatments, focusing on the processes involved in emotional disorders rather than relying solely on diagnostic labels.

Currently, to our knowledge, there are only two validation studies of the MEDI, in addition to the original validity study (Rosellini & Brown, 2019), conducted with Hispanic populations. One study was carried out with Spanish university students (Osma et al., 2021), and the other was conducted in a Spanish public mental health setting (Osma et al., 2023). The results of these studies demonstrate that a 9-factor solution fits the data well, as reported in the original article (χ2(1, 051) = 2,310.9, *p* < 0.001, RMSEA = 0.04, TLI = 0.92, CFI = 0.94, SRMR = 0.02) (Rosellini & Brown, 2019). Additionally, the two Spanish studies confirm the 9-factor solution with non-clinical Spanish students (χ2/df = 1.59, CFI = 0.865; RMSR = 0.074; RMSEA = 0.051) and clinical Spanish population (χ2/df = 1.69, CFI = 0.868; SRMR = 0.066; RMSEA = 0.047) (Osma et al., 2021, 2023).

Further research into the validation of the MEDI in both clinical and non-clinical populations is warranted. Recently, in Colombia, there has been a growing interest in researching the efficacy (Castro-Camacho et al., 2023), effectiveness (Castro-Camacho et al., 2018, 2019), prevention (Castro-Camacho et al., 2022), and dissemination of transdiagnostic treatments (Zarate‐Guerrero et al., 2022). Given the transdiagnostic structure of the MEDI, having a validated version for the Colombian population would be immensely beneficial. Specifically, validation in a non-clinical population would enable a focus on prevention and early detection of risks associated with the development of emotional disorders, leading to more dimensional and personalized prevention programs (Craske, 2017).

Considering this background, the present study aims to validate the MEDI in the context of a non-clinical Colombian population to identify transdiagnostic individual characteristics. Initially the study involves adapting the MEDI to the Spanish language, including cultural adaptation for the Colombian population. Subsequently, it examines the psychometric characteristics within a non-clinical sample of Colombian university students. This study aims to replicate the original statistical analysis performed by the creators of the MEDI (Rosellini & Brown, 2019), that is to compare the 7, 8 and 9 factor structure of the MEDI. Additionally, it seeks to compare the results with those obtained from the Spanish non-clinical population study.

## Methods

### Participants and sample characteristics

The sample consisted of 935 students who consented to participate in the study and completed the screening battery. Among these, 111 were excluded because they were receiving psychological or psychiatric treatment concurrently with the study, and an additional 16 were excluded from the analysis due to incomplete questionnaire responses. The final sample included 808 participants, with a mean age of 21.9 years (*SD* = 4.65, range = 18 – 37), who were undergraduate students at three different private universities in Bogotá, Colombia. The sample predominantly comprised women (*N* = 512) and men (*N* = 296). The majority of participants were from *Universidad de los Andes* (*N* = 400), with the remainder divided between *Universidad Areandina* (*N* = 270) and *Universidad Sergio Arboleda* (*N* = 138). Regarding academic disciplines most participants were enrolled in Social Sciences departments (*N* = 480), followed by Humanities and Human Arts (*N* = 130), and Engineering programs (*N* = 198). Even though

 no exact socio-economic status (SES) information was collected, it can be inferred that each of the universities represents different SES:high-income, middle-income, and low-income students.

### Instruments

The Multidimensional Emotional Disorder Inventory (MEDI) consists of 49 items, originally formulated by Rosellini in 2013. Respondents evaluate these items using a Likert-type scale, ranging from 0 (not at all characteristic of me) to 8 (totally characteristic of me).

Validation Process to validate the MEDI, the original instrument (Rosellini et al., 2013; Rosellini and Brown, 2019) underwent translation into Spanish, with necessary cultural adaptations. Two proficient English researchers and one supervisor oversaw the translation process, resolving any discrepancies. Subsequently, the translated scale was sent to a native Spanish speaker, fluent in English, who was a member of the Center of Anxiety and Related Disorders (CARD) at Boston University, for back-translation and comparison against the original version. No significant differences were found between the original (Rosellini, 2013) and the Spanish version (Osma et al., 2021) and the obtained version. Appendix A includes the Spanish-Colombian adaptation of the MEDI. Upon approval of the instrument's translation by the authors, it was administered to the participants. Ethical approval was obtained, and all participants provided informed consent.

### Procedure

The participants in this study were recruited from three different private universities in Bogotá, Colombia, where they were pursuing their university studies. Inclusion criteria mandated that participants be at least 18 years old, enrolled as university students at the time of assessment, and willing to voluntarily participate by signing an informed consent form. The sole exclusion criterion was undergoing psychological or psychiatric treatment at the time of assessment.

Collaborating professors from various universities in Bogotá, Colombia assisted with participant recruitment. They disseminated recruitment messages to their undergraduate students via email, social media platforms, and campus advertisements. A hyperlink directing students to the online survey platform Qualtrics was provided for study access, and initial screening for student eligibility was conducted upon accessing the online survey. Informed consent procedures were administered electronically. Once eligibility was confirmed and consent obtained, participants were instructed to complete the demographic questionnaire and the MEDI instrument. The entire process typically required 20 min to complete. Participation in the study was voluntary, anonymous, and did not involve any monetary compensation. The research protocol received approval from the Research and Ethics Committee of Universidad de los Andes.

## Data analysis

Data analysis followed the methodology employed by the original researchers (Rosellini & Brown, 2019) and was conducted using M-Plus 7.1 (Muthén & Muthén, 2013). Robust maximum likelihood minimization functions were utilized to address issues related to non-normality and missing data.

To examine the structural integrity of the MEDI instrument within our sample, exploratory structural equation modeling (ESEM) was employed (Marsh et al., 2014). ESEM was chosen over traditional exploratory factor analysis (EFA) due to its ability to detect localized areas of tension within the model, such as standardized residuals and modification indices. Importantly, an ESEM model featuring a fully saturated factor-loading matrix devoid of localized areas of strain is statistically akin to a traditional maximum likelihood EFA with a fully saturated factor-loading matrix. Confirmatory factor analysis (CFA) was not employed due to the complex nature of the MEDI (49 items loading on 9 factors) and the potential for cross-loadings and error covariances, which would render a stringent measurement model unrealistic. Previous research indicates that CFA for measures with 50 + items or five or more factors is unlikely to yield a good fit (Marsh et al., 2014). Given the substantial comorbidity found in emotional disorder patient samples (Brown et al., 2001), it was anticipated that numerous items would exhibit cross-loadings ≥ 0.30.

Model adequacy was assessed using multiple goodness-of-fit indices, including the root mean squared error of approximation (RMSEA) and its close-fit test (C-Fit), the Tucker Lewis Index (TLI), the comparative fit index (CFI), and the standardized root mean square residual (SRMR). These indices evaluated different facets of model fit, including absolute fit, parsimonious fit, and fit relative to the null model (Brown, 2015). Generally accepted thresholds for adequate model fit include an RMSEA near or below 0.06, a C-Fit above 0.05, TLI and CFI values close to or above 0.95, and an SRMR near or below 0.08 (Hu & Bentler, 1999).

## Results

This study aimed to replicate the analysis conducted in the original MEDI validation study (Rosellini & Brown, 2019) using a non-clinical, community-based sample of 808 students from three private universities in Bogota, Colombia. The Colombian version of the MEDI was assessed by comparing three exploratory structural equation modeling (ESEM) solutions: 7, 8, and 9 factor models, consistent with the analysis performed by Rosellini and Brown (2019). However, upon closer examination, while all potential solutions demonstrated an overall model fit, none of the 7, 8, or 9 factor models appeared to adequately organize dimensions compared to the original MEDI structure and theoretical framework (Refer to Table 1). Notably, some solutions featured an isolated dimension, with only item 46 present ("Although I know they are unrealistic, I have thoughts about losing control of my actions") (see Table 2). Item 46 showed weak correlations with other items and posed translation challenges, leading to its exclusion. The 48-item ESEM solution, excluding item 46, demonstrated an acceptable model fit and a more coherent item organization, as depicted in Table 3.

**Table 1:** Confirmatory factor analysis – Model fit

	7 factors 48 items	7 factors 49 items	**8 factors 48 items**	8 factors 49 items	9 factors 48 items	9 factors 49 items
Chi-Square Test of Model Fit (χ2)	1493.64*	1581.69*	1341.19*	1385.70*	1326.04*	1347.94*
Degrees of Freedom	813	854	**772**	812	732	771
RMSEA	0.04	0.04	**0.04**	0.04	0.04	0.04
CFI	0.91	0.91	**0.92**	0.93	0.92	0.93
TLI	0.88	0.87	**0.89**	0.89	0.88	0.89
SRMR	0.02	0.02	**0.02**	0.02	0.02	0.02

The table shows model fit statistics of 7,8 and 9 factors of the 48 and the 49 items ESEM solution. * = *p* > 0.0001. *RMSEA* Root mean square error of approximation, *CFL* Comparative fit index, *TLI* Tucker lewis index, *SRMR* Standard root mean square residual. In bold the final model selected

**Table 2:** Factor Loadings from 9-Factor ESEM solution (49 items)

Item (#)	Spanish—Colombian Translation	Neuro	PosT	Depr	SocAn	SomA	AutAr	Traum	Intru Cog	Avoid
Easily Upset (1)	*Irritación cosas triviales*	**0.29**	-0.16	0.07	0.10	0.00	0.04	0.13	0.02	-0.05
Easily laughs (2)	*Facilidad al reirse*	0.12	**0.41**	0.08	0.01	-0.08	0.06	-0.09	-0.13	0.10
Disappointed in self (3)	*Decepción de si mismo*	0.15	-0.14	**0.63**	0.08	-0.00	-0.05	0.02	-0.08	0.02
Experiencing breathlessness (4)	*Sensación ahogo, falta de aire*	0.08	-0.05	0.16	-0.06	0.12	**0.44**	0.11	-0.03	0.07
Odd thoughts (5)	*Pensamientos Raros*	-0.01	0.07	**0.38**	0.07	0.09	-0.00	0.07	0.13	0.08
Fears physical sensations (6)	*asusta sentir sensaciones físicas inesperadas*	0.04	0.00	0.00	0.14	**0.31**	0.10	0.13	0.00	0.14
Uncomfortable mingling (7)	*incomodidad hablando personas en eventos sociales*	-0.01	-0.05	0.03	**0.78**	0.05	-0.07	0.04	-0.13	0.01
Thinking about horrific experiences (8)	*parar de pensar cosas horribles vividas o vistas*	0.06	-0.01	0.16	0.04	-0.05	0.02	**0.64**	-0.11	0.11
Distraction coping (9)	*distracción para manejar pensamientos, sentimientos o imágenes desagradables*	0.07	0.15	0.10	0.05	-0.04	0.05	0.06	-0.02	**0.35**
Always been worrier (10)	*tendencia a preocuparse por todo*	**0.74**	0.03	0.02	0.05	0.05	0.03	-0.01	-0.02	-0.00
Feel sad (11)	*sentimiento triste y melancólico*	0.19	-0.09	**0.41**	0.08	0.05	0.11	0.11	-0.00	0.07
Unpleasant thoughts/images (12)	*llegan a mente imágenes, pensamientos, recuerdos desagradables*	0.12	-0.10	0.07	-0.02	0.01	0.08	**0.57**	0.09	0.17
Feeling trembling/shaky (13)	*tremulo o tembloroso*	0.03	-0.01	0.18	0.03	0.05	**0.47**	0.28	-0.13	0.03
Uncomfortable center of attention (14)	*incomodidad en situaciones centro atención*	0.06	-0.15	-0.01	**0.56**	-0.05	0.05	0.12	-0.23	0.15
Avoids upsetting places/things (15)	*dificultad lugares o cosas que hacen sentir mal*	0.13	0.04	0.03	0.18	0.10	0.03	-0.11	0.03	**0.47**
Poor stress coping (16)	*trabajo controlando estrés*	**0.54**	-0.05	-0.01	0.02	0.06	0.09	0.06	0.04	0.09
Optimistic person (17)	*persona optimista*	-0.14	**0.69**	-0.11	-0.03	0.05	0.07	-0.07	0.00	0.11
Sudden rushes fear (18)	*picos repentinos de miedo*	0.17	0.08	0.11	0.08	0.06	**0.27**	0.25	0.07	0.02
Worry about health (19)	*preocupación por la salud*	0.02	0.04	0.06	0.05	**0.69**	0.18	-0.04	-0.05	-0.10
Disturbing dreams of past events (20)	*sueños molestos sobre eventos horrorosos del pasado*	0.06	0.14	-0.04	0.04	0.07	0.04	**0.76**	-0.09	-0.13
Inappropriate/nonsensical thoughts (21)	*entran en la mente pensamientos inapropiados que no se pueden quitar*	0.13	-0.07	0.06	0.11	0.01	-0.03	**0.49**	0.21	0.1
Anxious with strangers (22)	*ansieadad ante personas desconocidas*	0.06	0.08	0.13	**0.72**	0.08	0.01	-0.02	0.09	-0.05
Carries protective objects (23)	*portar cosas para protegerse de situaciones o sentimientos incomodos*	-0.14	0.13	0.27	**0.30**	0.05	0.20	0.08	0.10	0.05
Cheerful and happy person (24)	*persona alegre y jovial*	-0.04	**0.74**	-0.13	-0.10	-0.03	0.08	0.02	-0.01	0.09
Loss of interest (25)	*pérdida del interés por actividades que se disfrutan*	0.09	-0.03	**0.39**	-0.09	0.06	0.12	0.16	0.03	-0.00
Felt dizzy/lightheaded/faint (26)	*sensación de mareos,vertigo,desmayo*	0.14	0.05	0.04	-0.07	0.26	**0.44**	0.02	0.00	0.00
Gets rid of unpleasant feelings (27)	*quitarse sentimientos desagradables*	0.11	-0.06	0.09	-0.07	0.20	-0.04	0.25	0.16	**0.30**
Preoccupied by illnesses (28)	*preocupación por enfermedades*	0.03	-0.07	-0.00	-0.01	**0.90**	-0.04	0.01	0.00	0.03
Intrusive images of past trauma (29)	*entran imágenes de eventos pasados traumáticos de forma inesperada*	0.01	-0.07	-0.05	-0.00	0.08	0.02	**0.83**	-0.01	-0.02
Actions driven by thoughts (30)	*acciones a menudo impulsadas por pensamientos o imágenes que no quiero tener*	-0.06	0.01	0.20	0.10	-0.01	0.10	**0.56**	0.08	-0.01
Tries to suppress upsetting thoughts (31)	*evitar pensar en cosas molestas*	0.03	0.23	0.08	0.00	0.07	0.02	-0.05	-0.00	**0.47**
More keyed up than average (32)	*nervioso y tenso de lo normal*	**0.25**	-0.07	0.19	0.18	0.08	0.17	-0.00	0.13	0.04
Always motivated (33)	*motivación emprender cosas nuevas*	0.07	**0.70**	0.09	0.03	0.01	-0.15	0.04	0.03	-0.19
Avoids feared objects (34)	*no contacto cosas que dan miedo*	0.02	0.05	0.04	0.21	0.08	0.01	0.07	-0.11	**0.33**
Feelings hurt easily (35)	*persona a quien hieren sentimientos facil*	**0.34**	0.01	-0.08	0.17	-0.06	0.06	0.15	0.08	0.17
Satisfied when finishing jobs (36)	*sensación bienestar terminar trabajo*	0.20	**0.50**	-0.05	-0.06	-0.00	-0.20	0.03	0.00	0.13
Nothing to look forward to (37)	*nada ilusiona*	-0.18	-0.05	**0.56**	0.06	0.00	0.01	-0.01	0.04	0.03
Closely monitor health (38)	*atención a salud por miedo a enfermarse*	-0.05	0.11	-0.08	-0.01	**0.73**	-0.07	0.06	-0.00	0.05
Feels like reliving trauma (39)	*sensación de revivir horribles eventos del pasado*	-0.04	0.01	0.08	0.02	0.01	0.06	**0.68**	0.08	0.01
Unacceptable thoughts/images (40)	*pensamientos o imágenes inaceptables*	0.03	-0.02	0.15	-0.02	0.07	-0.06	**0.54**	0.23	0.13
Nervous when talking to others (41)	*nervios hablar personas*	0.06	0.01	-0.02	**0.83**	-0.02	0.03	-0.01	-0.04	0.04
Routine actions taken to cope (42)	*actos rutinarios para afrontar sentimientos o situaciones incómodas*	0.02	0.06	0.07	0.22	0.19	0.10	0.06	0.13	**0.22**
Life not worth living (43)	*pensando que no vale la pena vivir*	0.04	-0.02	**0.61**	-0.02	-0.04	0.09	0.09	0.09	0.03
High resting heat rate (44)	*corazón late rapido incluso no actividad física*	0.16	-0.04	-0.06	0.09	0.01	**0.57**	0.06	0.17	0.04
Believes has undiagnosed illness (45)	*podría tener enfermedad no diagnosticada*	0.09	-0.05	0.10	0.02	**0.38**	0.17	-0.00	0.28	-0.01
Aunque no es real pienso que puedo perder el control de mis actos (46)	*Unrealistic fear of losing control*	0.05	0.00	0.16	0.05	-0.02	0.15	0.24	**0.41**	0.07
Nervous in social situations (47)	*siento nervios situaciones sociales*	-0.00	-0.04	-0.04	**0.92**	-0.02	0.00	-0.00	0.11	-0.02
Distressed by trauma reminders (48)	*enojo altero cuando me acuerdo cosas horribles que he vivido o visto*	0.06	-0.05	-0.02	0.15	-0.00	0.09	**0.42**	0.31	0.03
Fears prevent day-to-day tasks (49)	*miedos no dejan realizar tareas cotidianas*	-0.04	-0.02	0.10	**0.34**	0.09	0.07	0.17	0.18	0.15

Completely standardized factor loadings are presented. Exploratory structural equation modeling was conducting with robust maximum likelihood estimation and quartimin rotation. Factor loadings ≥|.30| are bolded. *ESEM* Exploratory structural equation modeling, *Tra/IC* Traumatic re-experiencing/Intrusive cognitions, *Neuro* Neurotic temperament, *SocAn* Social anxiety, *PosT* Positive temperament, *SomA* Somatic anxiety, *Depr* Depressed mood, *AutAr* Autonomic arousal, *Avoid* Avoidance

To improve the model fit of the 48-item ESEM solution, we examined modification indices and standardized residuals. Specifically, items 40 ("I have thoughts or images that I find unacceptable") and 21 ("Inappropriate or nonsensical thoughts enter my mind that are difficult to dismiss") showed strong correlations and theoretical similarities. Consequently, we developed a model incorporating their modification indices (MI). This final 48-item ESEM solution with MI demonstrated a robust fit to the data: χ2(771) = 1316.706, *p* < 0.001, RMSEA = 0.043, TLI = 0.90, CFI = 0.932, and SRMR = 0.027. Most items exhibited substantial loadings (≥ 0.30), as outlined in Table 3. Additionally, a parallel analysis conducted in Mplus suggested that a model with five to eight factors could be appropriate, further supporting our selection of an eight-factor model.

**Table 3:** Factor Loadings from 8-Factor ESEM solution (48 items)

Item (#)	Spanish—Colombian Translation	Neuro	PossA	Depr	SocAn	SomA	AutAr	Trau/IC	Avoid
Easily Upset (1)	*Irritación cosas triviales*	**0.22**	-0.18	0.06	0.12	0.00	0.12	0.15	-0.05
Easily laughs (2)	*Facilidad al reirse*	0.20	**0.45**	0.14	0.03	-0.07	0.02	-0.09	-0.10
Disappointed in self (3)	*Decepción de si mismo*	0.15	-0.15	**0.65**	0.09	0.01	-0.06	0.02	-0.04
Experiencing breathlessness (4)	*Sensación ahogo, falta de aire*	0.09	-0.03	0.17	-0.06	0.09	**0.47**	0.09	-0.00
Odd thoughts (5)	*Pensamientos Raros*	-0.02	0.08	**0.39**	0.07	0.08	0.01	0.1	0.09
Fears physical sensations (6)	*asusta sentir sensaciones físicas inesperadas*	0.10	0.04	0.03	0.15	**0.30**	0.08	0.14	0.04
Uncomfortable mingling (7)	*incomodidad hablando personas en eventos sociales*	0.03	-0.04	0.04	**0.79**	0.04	-0.11	0.02	-0.08
Thinking about horrific experiences (8)	*parar de pensar cosas horribles vividas o vistas*	0.12	0.01	0.18	0.04	-0.05	-0.02	**0.62**	0.01
Distraction coping (9)	*distracción para manejar pensamientos, sentimientos o imágenes desagradables*	0.19	0.22	0.15	0.08	-0.03	0.00	0.06	**0.17**
Always been worrier (10)	*tendencia a preocuparse por todo*	**0.57**	-0.00	0.03	0.11	0.09	0.13	0.03	-0.01
Feel sad (11)	*sentimiento triste y melancólico*	0.17	-0.10	**0.41**	0.09	0.05	0.14	0.11	0.04
Unpleasant thoughts/images (12)	*llegan a mente imágenes, pensamientos, recuerdos desagradables*	0.13	-0.08	0.08	-0.01	0.01	0.09	**0.59**	0.20
Feeling trembling/shaky (13)	*tremulo o tembloroso*	0.06	0.00	0.20	0.03	0.03	**0.42**	0.26	-0.08
Uncomfortable center of attention (14)	*incomodidad en situaciones centro atención*	0.17	-0.1	0.02	**0.57**	-0.04	-0.02	0.08	-0.04
Avoids upsetting places/things (15)	*dificultad lugares o cosas que hacen sentir mal*	0.25	0.12	0.09	0.21	0.11	0.04	-0.12	**0.31**
Poor stress coping (16)	*trabajo controlando estrés*	**0.45**	-0.06	0.00	0.06	0.08	0.19	0.09	0.08
Optimistic person (17)	*persona optimista*	-0.10	**0.72**	-0.08	-0.02	0.04	0.04	-0.08	0.05
Sudden rushes fear (18)	*picos repentinos de miedo*	0.11	0.07	0.11	0.1	0.05	**0.31**	0.27	0.03
Worry about health (19)	*preocupación por la salud*	-0.00	0.03	0.07	0.05	**0.68**	0.20	-0.04	-0.15
Disturbing dreams of past events (20)	*sueños molestos sobre eventos horrorosos del pasado*	0.04	0.12	-0.05	0.04	0.07	0.01	**0.78**	-0.18
Inappropriate/nonsensical thoughts (21)	*entran en la mente pensamientos inapropiados que no se pueden quitar*	0.06	-0.08	0.04	0.13	0.01	0.06	**0.50**	0.18
Anxious with strangers (22)	*ansieadad ante personas desconocidas*	-0.01	0.05	0.11	**0.74**	0.08	0.04	-0.00	0.00
Carries protective objects (23)	*portar cosas para protegerse de situaciones o sentimientos incomodos*	-0.18	0.13	0.26	**0.29**	0.03	0.21	0.08	0.10
Cheerful and happy person (24)	*persona alegre y jovial*	-0.01	**0.78**	-0.09	-0.09	-0.03	0.05	0.03	0.00
Loss of interest (25)	*pérdida del interés por actividades que se disfrutan*	0.05	-0.04	**0.40**	-0.09	0.07	0.13	0.19	-0.03
Felt dizzy/lightheaded/faint (26)	*sensación de mareos,vertigo,desmayo*	0.08	0.05	0.04	-0.07	0.24	**0.52**	0.02	-0.04
Gets rid of unpleasant feelings (27)	*quitarse sentimientos desagradables*	0.10	-0.03	0.10	-0.07	0.20	0.00	0.26	**0.39**
Preoccupied by illnesses (28)	*preocupación por enfermedades*	0.03	-0.07	-0.00	-0.00	**0.91**	-0.03	0.01	0.042
Intrusive images of past trauma (29)	*entran imágenes de eventos pasados traumáticos de forma inesperada*	0.01	-0.07	-0.05	-0.01	0.07	-0.00	**0.85**	-0.03
Actions driven by thoughts (30)	*acciones a menudo impulsadas por pensamientos o imágenes que no quiero tener*	-0.11	0.00	0.18	0.09	-0.02	0.11	**0.57**	0.04
Tries to suppress upsetting thoughts (31)	*evitar pensar en cosas molestas*	0.16	0.32	0.13	0.03	0.07	0.01	-0.07	**0.31**
More keyed up than average (32)	*nervioso y tenso de lo normal*	0.15	-0.10	0.17	0.20	0.09	**0.24**	0.03	0.13
Always motivated (33)	*motivación emprender cosas nuevas*	-0.02	**0.61**	0.05	0.03	0.02	-0.13	0.07	-0.11
Avoids feared objects (34)	*no contacto cosas que dan miedo*	0.14	0.12	0.09	**0.23**	0.08	-0.02	0.03	0.15
Feelings hurt easily (35)	*persona a quien hieren sentimientos facil*	**0.30**	0.01	-0.08	0.19	-0.05	0.11	0.17	0.21
Satisfied when finishing jobs (36)	*sensación bienestar terminar trabajo*	0.21	**0.50**	-0.03	-0.04	0.00	-0.17	0.04	0.09
Nothing to look forward to (37)	*nada ilusiona*	-0.17	-0.05	**0.57**	0.05	-0.00	-0.02	-0.00	0.01
Closely monitor health (38)	*atención a salud por miedo a enfermarse*	-0.03	0.12	-0.08	-0.01	**0.72**	-0.07	0.05	0.04
Feels like reliving trauma (39)	*sensación de revivir horribles eventos del pasado*	-0.07	0.02	0.08	0.01	0.00	0.07	**0.70**	0.04
Unacceptable thoughts/images (40)	*pensamientos o imágenes inaceptables*	-0.01	-0.00	0.15	-0.01	0.07	0.00	**0.56**	0.22
Nervous when talking to others (41)	*nervios hablar personas*	0.05	0.02	-0.02	**0.85**	-0.02	0.04	-0.03	0.01
Routine actions taken to cope (42)	*actos rutinarios para afrontar sentimientos o situaciones incómodas*	-0.00	0.08	0.06	0.23	0.18	0.14	0.06	**0.31**
Life not worth living (43)	*pensando que no vale la pena vivir*	-0.02	-0.04	**0.60**	-0.02	-0.04	0.12	0.11	0.09
High resting heat rate (44)	*corazón late rapido incluso no actividad física*	0.08	-0.04	-0.06	0.10	-0.00	**0.64**	0.09	0.07
Believes has undiagnosed illness (45)	*podría tener enfermedad no diagnosticada*	-0.03	-0.07	0.09	0.03	**0.36**	0.27	0.05	0.13
Nervous in social situations (47)	*siento nervios situaciones sociales*	-0.05	-0.06	-0.05	**0.93**	-0.02	0.01	0.01	0.03
Distressed by trauma reminders (48)	*enojo altero cuando me acuerdo cosas horribles que he vivido o visto*	-0.02	-0.06	-0.02	0.16	-0.00	0.15	**0.48**	0.17
Fears prevent day-to-day tasks (49)	*miedos no dejan realizar tareas cotidianas*	-0.08	-0.00	0.09	**0.35**	0.07	0.10	0.18	0.25
α		0.72	0.75	0.8	0.87	0.81	0.84	0.92	0.69

Completely standardized factor loadings are presented. Exploratory structural equation modeling was conducting with robust maximum likelihood estimation and quartimin rotation. Factor loadings ≥|.30| are bolded. *ESEM* Exploratory structural equation modeling, *Tra/IC* Traumatic re-experiencing/Intrusive cognitions, *Neuro* Neurotic temperament, *SocAn* Social anxiety, *PosT* Positive temperament, *SomA* Somatic anxiety, *Depr* Depressed mood, *AutAr* Autonomic arousal, *Avoid* Avoidance. Factor loadings (corrected item-test correlation). *α* Cronbach's alpha

### Factor and scale correlations and reliability

Table 4 displays the means, standard deviations, and correlations between all MEDI dimensions. Significant correlations were observed among all MEDI dimensions, as depicted in Table 4, except for the correlations between Positive Affect and Avoidance (*r* = 0.07, *p* = 0.133) and Positive Affect and Somatic Anxiety (*r* = 0.017, *p* = 0.739). As expected, Positive Affect showed negative correlations with the other phenotype dimensions, while Neurotic Temperament exhibited positive correlations with the other phenotype dimensions (excluding Positive Affect). Descriptive statistics indicated similar means across dimensions, consistent with expectations for a non-clinical population. .

**Table 4:** Correlations between MEDI dimension

MEDI factor	Trauma Intrusive Cognition	Neurotic Temperament	Social Anxiety	Positive Temperament	Somatic Anxiety	Depression	Autonomic Arousal	Avoidance
Mean	23.7	17.9	26.3	31.1	17.1	19.1	17.3	21.2
SD	15.9	7.19	13.9	7.84	9.3	9.7	10.4	8.4
TraumaIntruCog	1	.60^**^	.57^**^	-.18^**^	.50^**^	.67^**^	.72^**^	.55^**^
NeuroticTemperament		1	.57^**^	-.13^**^	.43^**^	.50^**^	.64^**^	.52^**^
SocialAnxiety			1	-.22^**^	.39^**^	.55^**^	.57^**^	.53^**^
PossitiveAffect				1	.01	-.27^**^	-.13^**^	.07
SomaticAnxiety					1	.40^**^	.60^**^	.48^**^
Depression						1	.63^**^	.47^**^
AutonomicArousal							1	.56^**^
Avoidance								1

Correlations, mean and standard deviation (SD) were derived from the exploratory structural equation modeling solution for the 48-item MEDI. Robust maximum likelihood estimation and quartimin rotation were used. **p < .001. *ESEM* Exploratory structural equation modeling, *MEDI* Multidimensional emotional disorder inventory

In summary, the MEDI validation underscores the robust reliability of most scales and confirms the model's goodness-of-fit as determined by ESEM analysis. An 8-dimension model with 48 items emerged as the most suitable solution, alligning with the majority of dimensions identified by Rosellini and Brown (2019) in the original MEDI validation. To enhance model fit, item number 46 was excluded from the analysis due to its poor alignment with the proposed dimensions.

## Discussion

The validation of the MEDI in the Colombian population represents a significant advancement in the fields of research and clinical psychology across Latin American countries. It introduces a culturally adapted dimensional tool capable of elucidating key transdiagnostic dimensions underlying emotional disorders. Moreover, it provides valuable insights into the fundamental emotional processes inherent in a non-clinical population, within the confines of their normative parameters and factor structure.

While the need for replication is evident, the definitive 48-item eight-factor ESEM solution highlights a concise organization of items within their respective dimensions. Of particular interest is the significant correlation and factor loading shared among items within the Traumatic Re-experimentation and Intrusive Cognition dimension. This observation, distinct from both the original validation by Rosellini and Brown (2019) and the Spanish validation by Osma et al. (2021), offers a unique perspective. Regardless of the examined models (ranging from 7 to 9 factors) and the inclusion or exclusion of item number 46, a consistent pattern linking items within the Traumatic Re-experimentation dimension with Intrusive Cognitions persists.

There are two hypotheses proposed to explain this outcome. The first hypothesis suggests that the observed pattern could be attributed to linguistic similarities among items encompassing both dimensions. Essentially, items related to Traumatic Re-experimentation and Intrusive Cognitions primarily revolve around negative or threatening thoughts. It is plausible that, within this non-clinical population, the content and interpretation of items from these dimensions exhibited considerable overlap. Studies in Latin America indicate that while this population also acknowledges the occurrence of intrusive thoughts (Radomsky et al., 2014), discerning between obsessions and the emergence of intrusive memories or images evoking trauma is not straightforward (Kuneman, 2010). This challenge stems from crafting items that differentiate cyclical thought processes like rumination and worry from obsessive thoughts or images of traumatic re-experiencing (Pascual-Vera et al., 2019). The 8-factor model demonstrated that many items initially loading on the Traumatic Re-Experimentation dimension in the original instrument by Rosellini and Brown (2019) were reassigned in this model to the common dimension of intrusive cognition/traumatic re-experiencing, with items associated with physiological activation allocated to autonomic arousal and somatic anxiety. Despite limited studies discerning the qualities of intrusive thoughts beyond diagnostic criteria (e.g., obsessive–compulsive disorder vs. depression), another study indicated the presence of intrusive thoughts in samples of patients with OCD and depression, as well as patients with trauma and OCD (Orozco et al., 2021). Thus, there is significant scope for further investigation into instruments or items enabling differentiation among types of intrusive thoughts, encompassing repetitive thought loops (e.g., rumination and worry), obsessive thoughts, and images of traumatic re-experiencing.

The second hypothesis worth exploring concerns the potential accentuation of disparities between Intrusive Cognitions and thoughts associated with Traumatic Re-experimentation within a clinical sample affected by emotional disorders. This differentiation may not only manifest in divergent mean scores across MEDI dimensions but also in the elicitation of processes and symptoms specific to trauma-related disorders—a characteristic seldom encountered within a non-clinical population (Contractor et al., 2020). A study in Colombia examining the effects of a culturally adapted Unified Protocol on a heterogeneous clinical sample of emotional disorders demonstrated that indeed, the clinical population exhibits high scores on tests measuring symptoms associated with PTSD, particularly the PCL-5 (Castro-Camacho et al., 2023). Moreover, a case study also reported in this same study indicated that in vulnerable clinical populations (from low-income backgrounds), the presence of trauma is common due to typical conditions of insecurity and a history of internal armed conflict in Colombia (Castro-Camacho et al., 2018). Therefore, it is anticipated that in a non-clinical sample consisting of students from private universities in the capital city, traumatic experiences that may emerge in responses to the MEDI will be absent. In other words, it is likely that in the sample of this study, the distinction between intrusive thoughts and trauma-related thoughts is not attributable to characteristics of the selected sample. To validate this hypothesis, a second study is currently underway (Zárate-Guerrero et al., 2024, in prep) aiming to validate the MEDI in a clinical population.

Although the 9-factor model in the present study is supported by fit indices, the issue lies in the theoretical interpretation of the 9 factors. As mentioned in the results section, the 9-factor model with the 49-item solution resulted in only one dimension with a single item loading on it (item 46). Since it did not load on other dimensions nor did other items in this dimension, theoretically and psychometrically, it is more parsimonious to proceed with a model of 8 factors without that item (Rosellini & Brown, 2021). While a qualitative analysis of the items was not conducted to investigate the meaning attributed to them by individuals in the selected sample, during the construction of the Colombian version of the instrument, the phrasing of the item was noted to be unclear. When compared to the Spanish version, there are no major linguistic differences in the item; therefore, it may indicate a cultural difference in interpretation.

Another noteworthy finding, when contrasting the present study with both the original validation and the Spanish validation, revolves around the diminished factor loading of items within the avoidance dimension. Intriguingly, some of the Avoidance items' variance is captured by the Social Anxiety dimension. Furthermore, certain items that originally resided within the Avoidance dimension of the MEDI instrument have now found a place within the Social Anxiety dimension within the Colombian MEDI dataset (e.g., item 34 "Avoids feared objects"; item 23 "Carries protective objects"). This observation underscores the interconnectedness of core processes in emotional disorders. As Brown and Barlow (2009) initially postulated, the core processes underpinning emotional disorders are dimensional in nature rather than categorical. This allows for the interplay of certain processes that may share a higher-order core process dimension (e.g., Neurotic Temperament).

Regarding the other dimensions (Neurotic Temperament, Positive Temperament, Somatic Anxiety, Autonomic Arousal, Depressed Mood), it is worth noting that, overall, the dimensions within the original MEDI instrument bear a strong resemblance to the Colombian MEDI validation. Additionally, the MEDI dimensions exhibit robust reliability and validity, underscored by acceptable factor determinacies and composite reliabilities. Similarly, the intercorrelations among factors exhibit modest-to-moderate magnitudes, aligning largely with prior research (Rosellini, 2013; Rosellini & Brown, 2019). This is unsurprising, given the extensive body of literature elucidating the universality of high-order factors in emotional disorders (Brown & Barlow, 2009; Rosellini & Brown, 2015), signifying that anxiety, depression, and related disorders are underpinned by transdiagnostically distinguishable processes.

Ultimately, this study addresses one of the "next steps" outlined by Rosellini and Brown (2019), where validation with a non-clinical population was recommended. Specifically, a pivotal highlight of this study lies in its replication of the same statistical analyses conducted by the original authors. This constitutes the primary distinction between this validation and the Spanish validation study. While more data are requisite to further refine the MEDI dimensions within the Colombian population, and naturally, a replication of this analysis is imperative within a clinical sample, these findings represent an auspicious initial stride in the development and adaptation of dimensional instruments. These instruments serve not only to gauge clinical progress across psychological treatments but also to categorize clinical populations based on their transdiagnostic core processes.

### Limitations of the present study

This study is subject to several limitations, underscoring the need for further research to extend the validation of the MEDI instrument across diverse contexts. Additional psychometric validation is warranted, both with clinical samples and non-clinical, non-student samples that can better represent the heterogeneous characteristics of the Colombian population. A specific limitation to recognize was the sample used, as all the participants were non-clinical university students from private universities of one city of Colombia, influencing the generalization of the results. The 48-item ESEM solution, characterized by its 8-factor structure, requires replication across various community and clinical samples. In fact, one study performed by the same authors of this study is trying to collect clinical data in a similar 800 sample with the same MEDI-Colombian version to verify or unconfirmed the current structure. Moreover, it would be beneficial to conduct more test–retest analyses and assess convergent and discriminant validity in relation to other diagnostic and transdiagnostic validated measures. Finally, this article does not include information regarding how to interpret the scores nor percentile range and T-Scores for each dimension. It is important to include replicate this study with a larger clinical sample and add this scores so clinical psychologist can use the measure in the Colombian and Latin-American context with its own percentile and T-Scores.

## Conclusions

This paper delves into a topic of considerable significance within contemporary clinical psychology research: the validation of dimensional measures tailored for clinical applications. The validation process of the MEDI within the Colombian population signifies an initial stride towards the development of dimensional, transdiagnostic tools designed to evaluate the fundamental processes that underlie emotional disorders. Extensive replication studies involving large and diverse clinical samples are imperative to further establish the utility of this dimensional instrument. Such an instrument holds substantial promise for both clinicians and researchers by offering valuable insights into the transdiagnostic dimensions associated with emotional disorders. Without the MEDI, researchers would otherwise need to select and administer multiple disparate questionnaires to assess their specific areas of interest. Moreover, the MEDI has the potential to greatly benefit clinicians by targeting the transdiagnostic dimensions of interest and facilitating cognitive-behavioral treatment planning through functional analysis. In conclusion, this study provides support for the 48-item MEDI as an efficient and valid tool for assessing eight extensively studied emotional disorder traits and phenotypes.

## Data Availability

The datasets generated during and/or analyzed during the current study are available from the corresponding author on reasonable request.

## Inventario Multidimensional de Trastornos Emocionales (MEDI) Translation to spanish done by Santiago Zarate

Por favor indique el grado en el cual cada una de las siguientes afirmaciones es característica de usted o se aplica a usted, colocando un valor entre 0 y 8 en la columna de la izquierda, de acuerdo con la siguiente escala.

**Table:**

0	1	2	3	4﻿	5	6﻿	7	8
No Característico mi/no aplica para mi		Ligeramente Característico de mi/aplica ligeramente para mi		Un tanto Característico de mi/aplica un tanto para mi		Muy Característico de mi/aplica considerablemente para mi		Extremamente Característico de mi/aplica mucho para mi

_____ 1. Me irrito por cosas triviales.

_____ 2. No se necesita mucho para hacerme reír.

_____ 3. Estoy decepcionado de mí mismo.

_____ 4. He estado sintiendo sensación de ahogo o falta de aire.

_____ 5. Algunas personas pueden considerar que algunos de mis pensamientos son raros.

_____ 6. Me asusta sentir sensaciones físicas inesperadas.

_____ 7. Me siento incómodo hablando con personas en eventos sociales.

_____ 8. No puedo parar de pensar en cosas horribles que he vivido o visto.

_____ 9. Manejo los pensamientos, sentimientos o imágenes desagradables, tratando de distraerme.

_____ 10. Siempre he tendido a preocuparme por todo.

_____ 11. Me siento triste y melancólico.

_____ 12. Me llegan a la mente en contra de mi voluntad, imágenes, pensamientos o recuerdos desagradables.

_____ 13. Me he sentido trémulo o tembloroso.

_____ 14. Me siento incómodo en situaciones en las que soy el centro de atención.

_____ 15. Evito personas, lugares o cosas que me hacen sentir mal.

_____ 16. Me ha costado trabajo controlar el estrés.

_____ 17. Me considero una persona optimista.

_____ 18. He estado sintiendo picos repentinos de miedo.

_____ 19. Siento preocupación por mi salud.

_____ 20. Tengo sueños molestos acerca de eventos horrorosos que ocurrieron en el pasado.

_____ 21. Se me meten en la mente pensamientos inapropiados o absurdos que no me los puedo quitar.

_____ 22. Me siento ansioso cuando hay personas desconocidas.

_____ 23. Cargo conmigo ciertas cosas para protegerme de situaciones o sentimientos incómodos.

_____ 24. Me considero una persona alegre y jovial.

_____ 25. He perdido el interés por actividades que por lo general disfruto.

_____ 26. He sentido mareos, vértigo o que me voy a desmayar.

_____ 27. Haría casi lo que fuera por quitarme mis sentimientos desagradables

_____ 28. Estoy preocupado por las enfermedades.

_____ 29. Me entran imágenes de eventos pasados traumáticos de forma inesperada.

_____ 30. Mis acciones a menudo están impulsadas por pensamientos o imágenes que no quiero tener.

_____ 31. Si algo me molesta, hago todo lo posible para no pensar en eso.

_____ 32. Me siento más nervioso y tenso que el común de la gente.

_____ 33. Siempre me siento motivado por emprender tareas nuevas

_____ 34. No acepto estar en contacto con cosas que me dan miedo.

_____ 35. Soy el tipo de persona a quien le hieren los sentimientos con facilidad.

_____ 36. Me siento bien luego de terminar un trabajo.

_____ 37. Siento que no hay nada que me ilusione.

_____ 38. Le pongo mucha atención a mi salud porque me da miedo enfermarme.

_____ 39. A veces siento que estoy reviviendo horribles eventos de mi pasado.

_____ 40. Tengo pensamientos o imágenes que considero inaceptables.

_____ 41. Me dan nervios cuando hablo con otras personas.

_____ 42. Llevo a cabo ciertos actos rutinarios para poder afrontar sentimientos o situaciones incómodas.

_____ 43. He estado pensando que no vale la pena vivir.

_____ 44. A veces mi corazón late muy rápido incluso cuando no estoy teniendo actividad física.

_____ 45. Creo que podría tener alguna enfermedad que aún no ha sido diagnosticada.

_____ 46. Siento nervios en situaciones sociales.

_____ 47. Me enojo o altero cuando me acuerdo de las cosas terribles que he vivido o visto.

_____ 48. Mis miedos no me dejar realizar algunas tareas cotidianas.

## Abbreviations

MEDI: Multidimensional emotional disorder inventory
ESEM: Exploratory structural equation modeling
DSM: Diagnostical and statistical manual of mental disorders
ICD: International classification of disease
RDoC: Research domain criteria
HiTOP: Hierarchical taxonomy of psychopathology
NT: Neurotic temperament
PT: Positive temperament
DM: Depressed mood
AA: Autonomic arousal
SOM: Somatic anxiety
SEC: Social anxiety
IC: Intrusive cognitions
TRM: Traumatic re-experiencing and dissociation
AVD: Avoidance
EFA: Exploratory factor analysis
CFA: Confirmatory factor analysis
RMSE: Root mean squared error of approximation
SRMR: Standardized root mean square residual
C-Fit: Close-fit
TLI: Tucker lewis index
CFI: Comparative fit index
MI: Modification indices
